# Mesenchymal stem cell‐loaded cardiac patch promotes epicardial activation and repair of the infarcted myocardium

**DOI:** 10.1111/jcmm.13097

**Published:** 2017-02-28

**Authors:** Qiang‐li Wang, Hai‐jie Wang, Zhi‐hua Li, Yong‐li Wang, Xue‐ping Wu, Yu‐zhen Tan

**Affiliations:** ^1^ Department of Anatomy, Histology and Embryology Shanghai Medical School of Fudan University Shanghai China

**Keywords:** cardiac patch, mesenchymal stem cells, epicardium, epicardial‐derived cells, myocardial infarction, myocardial repair

## Abstract

Cardiac patch is considered a promising strategy for enhancing stem cell therapy of myocardial infarction (MI). However, the underlying mechanisms for cardiac patch repairing infarcted myocardium remain unclear. In this study, we investigated the mechanisms of PCL/gelatin patch loaded with MSCs on activating endogenous cardiac repair. PCL/gelatin patch was fabricated by electrospun. The patch enhanced the survival of the seeded MSCs and their *HIF‐1*α, *T*β*4*,* VEGF* and *SDF‐1* expression and decreased *CXCL14* expression in hypoxic and serum‐deprived conditions. In murine MI models, the survival and distribution of the engrafted MSCs and the activation of the epicardium were examined, respectively. At 4 weeks after transplantation of the cell patch, the cardiac functions were significantly improved. The engrafted MSCs migrated across the epicardium and into the myocardium. Tendency of *HIF‐1*α, *T*β*4*,* VEGF*,* SDF‐1 and CXCL14* expression in the infarcted myocardium was similar with expression *in vitro*. The epicardium was activated and epicardial‐derived cells (EPDCs) migrated into deep tissue. The EPDCs differentiated into endothelial cells and smooth muscle cells, and some of EPDCs showed to have differentiated into cardiomyocytes. Density of blood and lymphatic capillaries increased significantly. More c‐kit^+^ cells were recruited into the infarcted myocardium after transplantation of the cell patch. The results suggest that epicardial transplantation of the cell patch promotes repair of the infarcted myocardium and improves cardiac functions by enhancing the survival of the transplanted cells, accelerating locality paracrine, and then activating the epicardium and recruiting endogenous c‐kit^+^ cells. Epicardial transplantation of the cell patch may be applied as a novel effective MI therapy.

## Introduction

MI is a leading cause of death of the cardiovascular diseases in the developed and developing countries. The overall prevalence for MI is 2.8% in US adults. MI prevalence is 4.0% for men and 1.8% for women 1. According to investigation of the European Society of Cardiology, one in six men and one in seven women in Europe will die from MI 2. After occlusion of a coronary artery or its branch, necrosis of myocardium triggers local inflammation, scar formation and remodelling of the ventricular wall. The patients die of heart failure or arrhythmia finally. At present, there is no complete cure for MI. Percutaneous coronary intervention and pharmacotherapy using diuretics, angiotensin‐converting enzyme inhibitors and β‐adrenergic receptor antagonists have significantly reduced MI mortality. These therapies may improve cardiac functions to some degree; however, the lost cardiomyocytes cannot be regained. Although heart transplantation is often required at end‐stage heart failure, this therapy is limited by the shortage of organ donors and long‐term immune rejection.

Resent preclinical studies and clinical trails have shown that stems cell transplantation may be an effective way to improve cardiac function and attenuate adverse ventricular remodelling of the ischaemic myocardium 3, 4. There are several candidate stem cells for cardiac transplantation that have been recently evaluated 5, 6. Compared with cardiac progenitor cells, embryonic or induced pluripotent stem cells, mesenchymal stem cells (MSCs) have been considered a very promising cell population for cardiac transplantation because it is easily isolated from bone marrow or adipose tissue and exhibits low immunogenicity and cardiomyocyte, vascular smooth muscle and endothelial cell differentiation potential, although this cardiogenic potential remains controversial 7, 8. Cardiac cell therapy (*via* intracoronary or intracardial injection) is limited by poor engraftment and significant cell death after transplantation 9, 10. The cells may leak out of the injection sites and washout through venous shunts. Because of the hostile local environment, survival and differentiation of the transplanted cells are low. Additionally, injection of stem cells into myocardium has a considerable risk of cardiac perforation and dysrhythmia. Therefore, enhancing retention and survival of stem cells is an important goal for approaches of stem cell transplantation. Following MI, the composition of the cardiac extracellular matrix alters dynamically 11. Delivery of stem cells with natural materials such as fibrin and collagen creates a beneficial environment for engraftment and survival of the cells. Moreover, the biomaterials may stimulate angiogenesis and promote differentiation of stem cells 12. Behaviours of the transplanted stem cells may be induced or manipulated by inherent material properties (such as adhesiveness, stiffness, nanostructure or degradability) 13. However, intramyocardial injection of stem cells and hydrogel matrix almost has no effect on restricting ventricular dilation.

Cardiac patch fabricated with natural or synthetic materials has been considered a promising strategy for stem cell therapy of the infarcted myocardium. Application of the MSC‐loaded type I collagen patch over the epicardial surface at the infarct site enhances cell retention and improves post‐infarct remodelling 14. Cardiac and subcutaneous adipose tissue‐derived progenitor cells can differentiate into cardiomyocytes and endothelial cells when loading onto a fibrin patch 15. Three‐dimensional‐printed gelatin/hyaluronic acid patch carrying cardiac‐derived progenitor cells reduces adverse cardiac remodelling and preserves cardiac performance, and the matrix supports engraftment and survival of the cells 16. Our early study suggested that poly(ɛ‐caprolactone) (PCL)/gelatin patch may effectively restrict the expansion of the infarcted ventricular wall and that patch‐delivered bone marrow‐derived MSCs promote angiogenesis and repair of the infarcted myocardium 17. Taken together, these studies show that cardiac patch carrying stem cells (hereafter termed ‘cell patch’) promotes retention and survival of the engrafted stem cells and provides beneficial effects on restricting ventricular dilation. However, the underlying mechanisms responsible for the regeneration of the post‐infarct myocardium by the cell patch remain unclear.

In this study, we investigate effects of bone marrow‐derived MSC‐loaded PCL/gelatin patch on activating endogenous repairing potential after epicardial transplantation in rat and transgenic mouse MI models. Survival, viability and expression of paracrine factors of the cells seeded on the patch in hypoxic and serum‐deprived condition were examined. After the MSC‐loaded patch was transplanted onto the epicardium, angiogenesis, lymphangiogenesis, cardiomyogenesis and reduction in adverse ventricular remodelling were evaluated as well as changes in expression of paracrine factors in the infarcted myocardium. Furthermore, we assessed the activation of the epicardium and recruitment of endogenous c‐kit^+^ cells after transplantation of the cell patch. Here we report that transplantation of the cell patch enhances survival of the cells in the patch and release of paracrine factors by the transplanted cell and local tissue. Moreover, the cell patch promotes differentiation of the transplanted MSCs into endothelial cells and smooth muscle cells and presents potential of differentiation towards cardiomyocytes. It is worth noting that the cell patch effectively activates the epicardium and promotes differentiation of the activated epicardium‐derived cells (EPDCs) towards endothelial cells, vascular smooth muscle cells and cardiomyocytes. The epicardial cell patch also enhances recruitment of endogenous c‐kit^+^ cells for repair of the infarcted myocardium. Thus, we suggest that the cardiac cell patch has effective therapeutic effects *via* exogenous and endogenous mechanisms.

## Materials and methods

### Preparation of patches and MSC seeding

PCL/gelatin nanofibrous membrane was fabricated as described previously 17. Briefly, PCL and gelatin (1:1 at weight ratio) were dissolved in 1,1,1,3,3,3‐hexafluoro‐2‐propanol; then, the solution was electrospun and dried overnight under vacuum. The membrane was cut into 0.8 × 0.8 cm pieces as patches. After sterilization under UV light in a 24‐well plate, the patches were washed with PBS and then immersed in DMEM for 2 hrs before use. MSCs were isolated from bone marrow of male SD rats 18. All animals were obtained from the Medical Institute Animal Center of Fudan University. The investigation was permitted by the Law of the People's Republic of China on the Protection of Wildlife and approved by the Institutional Animal Care Committee of Fudan University. In experiments *in vitro*, the cells were uniformly seeded onto the patches at 1 × 10^4^ cells/patch. For tracing the transplanted cells, the cells were infected with lentiviruses carrying GFP (green fluorescent protein) expression cassette. Following cell seeding at 2 × 10^6^ cells/patch, the patches were incubated for 2 or 3 days. The cells growing on the patches were examined using a scanning electron microscope (SU8010; Hitachi, Tokyo, Japan).

### Treatment of the cells in hypoxic and serum‐deprived condition

To imitate hypoxic and ischaemic microenvironment, MSC‐seeded patch was placed in a sealed anoxia chamber containing 1% O_2_, 5% CO_2_ and 94% N_2_ and incubated in serum‐free medium. After incubation for 12 hrs, apoptosis of the cells was determined by EB/AO (ethidium bromide and acridine orange) staining. Cell viability was assessed by MTT assay. Expression of HIF‐1α (hypoxia‐inducible factor 1‐α), Tβ4 (thymosin β4), VEGF (vascular endothelial growth factor), SDF‐1 (stromal cell‐derived factor 1) and CXCL14 (chemokine (C‐X‐C motif) ligand 14) mRNAs in the cells was examined by qRT‐PCR (quantitative real‐time PCR) analysis. Primers used for amplification are listed in Table 1. Reaction conditions were 95°C for 30 sec., followed by 40 cycles at 95°C for 15 sec., at 54°C for 45 sec. and at 72°C for 45 sec.

**Table 1:** Sequences of primers

Symbol	Source	Version	Sequences	Length
*HIF‐1*α	Rat	NM_024359.1	(F)AAGTCTAGGGATGCAGCA	175 bp
			(R)CAAGATCACCAGCATCTAG	
*T*β*4*	Rat	NM_031136.1	(F)CAGCTCCTTCCAGCAACCAT	202 bp
			(R)AAGGCAATGCTCGTGGAATG	
*VEGF*	Rat	NM_001287114.1	(F)CGAGACGCAGCGACAAGGCA	171 bp
			(R)ACCTCTCCAAACCGTTGGCACG	
*SDF‐1*α	Rat	NM_022177.3	(F)GATTCTTTGAGAGCCATGTCGC	193 bp
			(R)AGTCCTTTGGGCTGTTGTGCTT	
*CXCL14*	Rat	NM_001013137	(F)TGTTCCCGGAAGGGGCCCAA	127 bp
			(R)GGCCGCGGTACCTGGACATG	
β*‐actin*	Rat	BC063166.1	(F)TGACCCAGATCATGTTTGAGA	186 bp
			(R)CAAGGTCCAGACGCAGGAT	

### Epicardial transplantation of the patches

Rat MI models were established according to the procedure described previously 19. Briefly, SD rats (200–250 g) were anaesthetized with ketamine (80 mg/kg) and xylazine (5–10 mg/kg) by peritoneal injection. The left anterior descending coronary artery (LAD) was ligated. One week after ligation, two rats died of heart failure. The rest survived 48 rats were randomly divided into sham operation (*n* = 6), control (*n* = 9), patch (*n* = 9), MSC (*n* = 12) and cell patch (*n* = 12) groups. In patch and cell patch groups, the patches were adhered onto the epicardium of the infarcted area with fibrinogen. Cell side of MSC‐seeded patch was down. In MSC group, 2 × 10^6^ cells (in 80 μl of PBS) were injected into peri‐infarct region at four sites. Injection of PBS in the infarcted rats and sham‐operated rats was used as control.

In Wt1^CreERT2/+^,R26^mTmG^ mouse (kindly provided by Dr. Bin Zhou, Institute for Nutritional Sciences, Shanghai Institutes for Biological Sciences, Chinese Academy of Sciences) MI models, transplantation of cell patches was performed as described previously. The mice were treated with intraperitoneal injection of tamoxifen (0.2 g/kg; Sigma‐Aldrich, Saint Louise, MO, USA) twice weekly for 3 weeks before MI was established. In the mice, a Cre‐modified oestrogen ligand‐binding domain (CreERT2) is knocked into the Wt1 locus. CreERT2 fusion protein recombinase activity is triggered by tamoxifen binding. There after, the *Wt1*‐expressing cells are labelled with membrane‐localized GFP and simultaneously inactivated expression of membrane‐localized dTomato fluorescent protein. These cardiac GFP^+^ cells, consisting of Wt1‐expressing cells and their descendants, are defined as EPDCs. For preparing cell patch, MSCs derived from bone marrow of male C57BL/6 mice were seeded on the PCL/gelatin membrane (0.6 **×** 0.6 cm).

### Echocardiographic examination

Echocardiograms were obtained before MI, at baseline (1 week after MI or before transplantation) and 4 weeks after transplantation. Rats were anaesthetized with ketamine (80 mg/kg) for echocardiographic examination. Left ventricular (LV) dimension and systolic function were evaluated using an echocardiographic machine (VisualSonics, Toronto, Ontario, Canada) with a 15 MHz linear transducer. LV dimension and thickness were measured with the American Society of Echocardiography leading‐edge technique, and the results are presented as the average of three measurements. After adequate two‐dimensional images were obtained, the M‐mode cursor was positioned to the parasternal long axis view at the level of the papillary muscles. LV end‐diastolic diameter (LVEDD) and LV end‐systolic diameter (LVESD) were measured from at least three consecutive cardiac cycles. LV end‐diastolic volume (LVEDV), LV end‐systolic volume (LVESV), index of ejection fraction (EF, LVEDV **−** LVESV/LVEDV × 100%), and fractional shortening (FS, LVEDD **−** LVESD/LVEDD × 100%) were measured for examination of systolic function. Two echocardiographers blinded to the experimental treatment acquired the images.

### Identification of the engrafted MSCs

The survival of the engrafted cells was evaluated by measuring the DNA copy of *Sry* gene on the Y chromosome of the cells by qRT‐PCR and GFP immunostaining. For qRT‐PCR, whole LV anterior wall was collected at 4 weeks after transplantation and digested with 1 ml of proteinase K solution for overnight. Hundred microlitres of the digested solution was used to isolate total DNA to count MSC number in each milligram. Total DNA from 1 × 10^6^ MSCs was used as standard after a serial dilution (10×). A standard curve was plotted as cycle number of *Sry* gene against log of cell number. The cell number in each milligram was calculated based on the cycle number of experimental rat DNA after qRT‐PCR. Assessments were performed in MSC and MSC‐seeded patch groups (three animals for each group) with SYBR^®^ Premix EX^™^ and a Rotor‐Gene 3000. Primer sequences were GGAGAGAGGCACAAGTTGGC (sense) and TCCCAGCTGCTTGCTGATC (anti‐sense). Reaction conditions were 50°C for 2 min., 95°C for 10 min., followed by 35 cycles at 95°C for 15 sec. and 60°C for 1 min.

### Expression of myocardial genes

To examine changes in expression of *HIF‐1*α, *T*β*4*,* VEGF*,* SDF‐1* and *CXCL14* in the infarcted cardiac tissue, total RNA was extracted from the whole LV wall at 7 days after transplantation in all groups (three animals for each group) and then assessed by qRT‐PCR. The methods were the same as described previously.

### Histological section of the heart

The hearts were fixed by perfusion with paraformaldehyde after echocardiographic examination, and transverse cryostat sections from upper, middle and lower parts of the hearts were obtained. Ten continuous sections from each part were collected and stained with Masson's trichrome and immunofluorescence, respectively. The infarct size was calculated as percentage of circumference of the infarct region in whole LV wall circumference. The LV wall thickness was measured at the minimum thickness region of the infarcted LV wall. Three sections in upper, middle and lower parts of each heart were, respectively, counted and averaged for six hearts in each group.

### Immunofluorescence staining

For labelling the transplanted cells, GFP immunostaining was performed. To assess differentiation of the transplanted cells towards endothelial cells and cardiomyocytes, co‐expression of CD31 or cTnT and GFP was determined by immunohistochemical double staining. Angiogenesis and lymphangiogenesis in the infarct and peri‐infarct regions were assessed by CD31 and LYVE‐1 immunostaining, respectively. Density of the microvessels or lymphatics in the infarct and peri‐infarct regions was examined by counting CD31‐positive or LYVE‐1‐positive structures from three independent sections of the middle part of the infarcted area. Five fields (20×) were randomly selected in each section (three sections per animal). Myocardial regeneration in the infarcted tissue was determined by expression of cTnT and Cx43.

To evaluate activation of the epicardial cells after transplantation of the cell patch, expression of Wt1 in rat hearts was examined with immunostaining. Wt1 was expressed in proepicardium and epicardium during the embryonic development and was regard as a marker of activation of the epicardial cells in adult 20. In transgenic mice, GFP^+^ cells were regarded as *Wt1*‐expressing cells. Differentiation of GFP^+^ cells towards endothelial cells, smooth muscle cells and cardiomyocytes was traced by double staining of GFP and CD31, α‐SMA or cTnT.

To assess mobilization of endogenous stem/progenitor cells in rat MI models, c‐kit immunostaining was performed. Double labelling of c‐kit and cTnT, α‐SMA or CD31 was used to evaluate differentiation of these cells towards cardiomyocytes, smooth muscle cells and endothelial cells. The endogenous c‐kit^+^ cells were distinguished with the transplanted MSCs by GFP and c‐kit double immunostaining.

The antibodies used for immunofluorescence staining are rabbit anti‐GFP antibody, mouse anti‐GFP antibody (1:200; Santa Cruz, Dallas, TX, USA), rabbit anti‐LYVE‐1 antibody (1:100; AngioBio, Del Mar, CA, USA), mouse anti‐CD31 antibody, mouse anti‐α‐SMA antibody, mouse anti‐cTnT and rabbit anti‐Cx43 (1:100; Abcam, Cambridge, MA, USA), rabbit anti‐Wt1 antibody and rabbit anti‐c‐kit antibody (1:100; Santa Cruz, USA). Goat antimouse (conjugated with Alexa Fluor 488) and goat anti‐rabbit (conjugated with Alexa Flour 594) IgG (1:400; Jackson, West Grove, PA, USA), or Dy‐Light 594 AffiniPure goat antimouse IgG (1:300; EarthOx, Millbrae, CA, USA) and FITC AffiniPure goat anti‐rabbit IgG (1:50; EarthOx).

### Statistical analysis

Data were expressed as mean ± S.D. SPSS 16.0 software (IBM, New York City, NY, USA) was used for all analyses. To analyse the data statistically, Student's *t*‐test and one‐way analysis of variance were performed with Scheffe's *post hoc* multiple comparison analysis.

## Results

### Biocompatibility of PCL/gelatin patch

**Figure 1:**
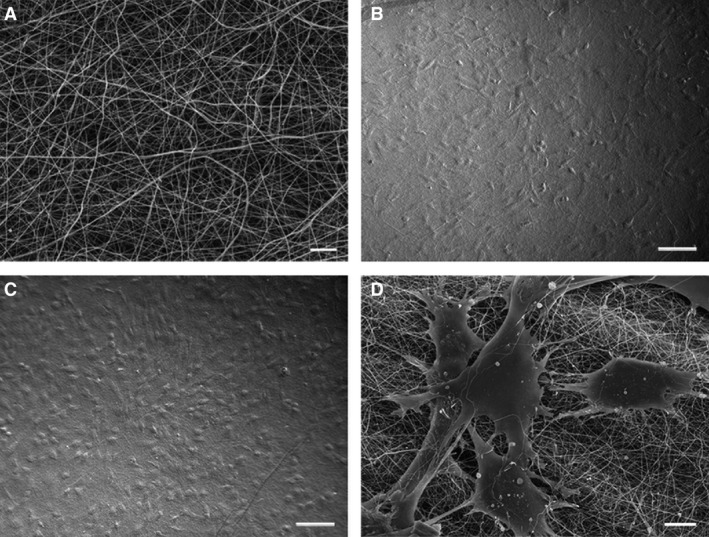
Architecture of the patch and growth of MSCs on the patch. (**A**) PCL/gelatin patch. The patch is porous and uniform nanofibrous. (**B** and **C**) MSCs growing on the patch for 24 hrs and 3 days, respectively. The cells spread well and are grown into monolayer (**C**). (**D**) the cells spreading on the nanofibrous patch. The cells spread well and connect to one another along the nanofibres. Some cells stretch into nanofibres. (**A** and **D**) scanning electron microscopic images. (**B** and **C**) phase contrast images. Scale bars represent 5 μm (**A**); 100 μm (**B** and **C**); and 10 μm (**D**).

The nanofibres of PCL/gelatin patch fabricated by electrospinning are uniform, and their diameter is about 240 nm. The size of pores in the patch is about 1.5 μm (Fig. 1A). At 24 hrs after seeding on the patch, MSCs adhered and spread well on the nanofibres (Fig. 1B). At 3 days after incubation, the cells growing on the nanofibrous patch proliferated and formed a dense monolayer (Fig. 1C), and the cells connected one another with pseudopods and stretch into nanofibres (Fig. 1D).

### Cytoprotective effect of the patch

**Figure 2:**
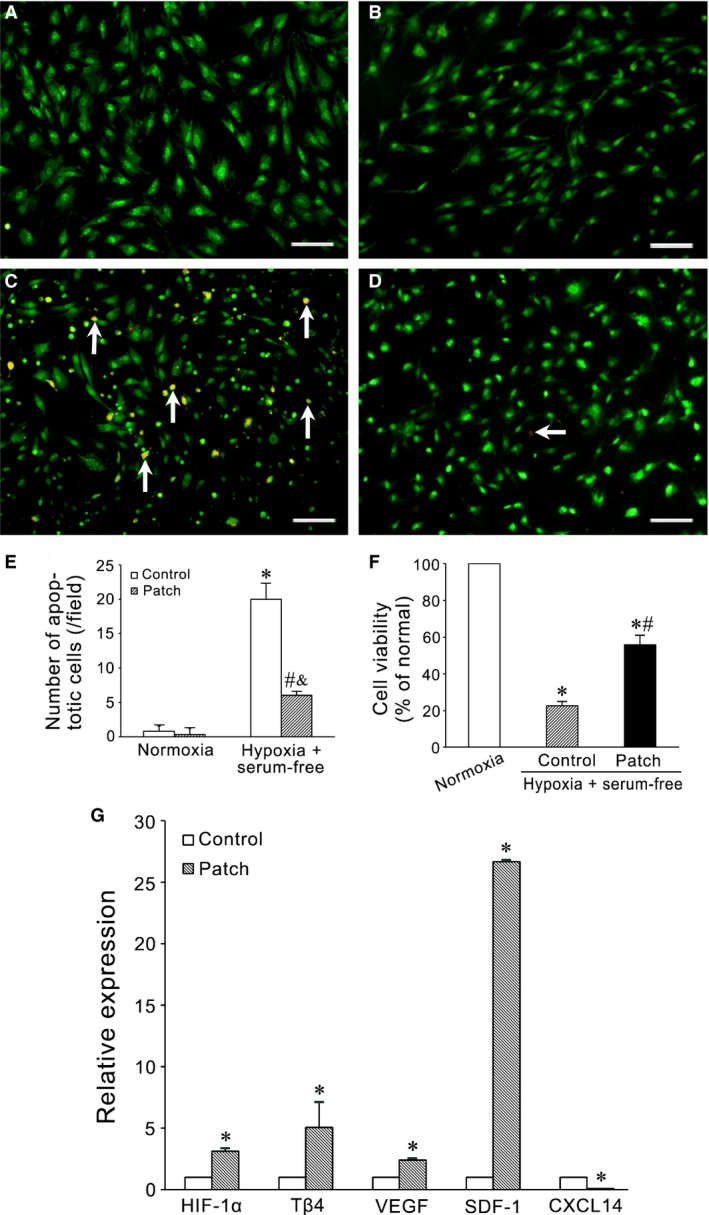
The survival, viability and gene expression of the cells seeded on the patches in hypoxia and serum deprivation. (**A–D**) the apoptotic cells identified with EB/AO staining. MSCs were seeded in petri dish (**A** and **C**) or on PCL/gelatin patch (**B** and **D**) and incubated for 12 hrs in the condition of normoxia (**A** and **B**) or hypoxia (1% O_2_, 5% CO_2_ and 94% N_2_) and serum‐free (**C** and **D**). In the condition of hypoxia and serum‐free, the apoptotic cells in patch group are less than that in control group. Scale bars: 100 μm. (**E**) statistical results of the apoptotic cells. *P < 0.01 *versus* control group in normoxic condition; ^#^ *P* < 0.01 *versus* patch group in normoxic condition; ^&^ *P* < 0.01 *versus* control group in hypoxic and serum‐free condition. (**F**) statistical results of cell viability. Compared with control group, cell viability increases in patch group. *P < 0.01 *versus* normal condition group (100%); ^#^ *P* < 0.01 *versus* control group in hypoxic and serum‐free condition. (**G**) the expression of *HIF‐1*α, *T*β*4*,* VEGF*,* SDF‐1* and *CXCL14* in MSCs. In patch group, the levels of *HIF‐1*α, *T*β*4*,* VEGF* and *SDF‐1* expression are higher. The level of *CXCL14* expression in patch group is lower than that in control group. *P < 0.05 *versus* control group.

The survival, viability and cytokine expression of MSCs seeded on PCL/gelatin patch were assessed with EB/AO staining, MTT assay and qRT‐PCR, respectively. The cell patches were treated with hypoxia and serum deprivation for 12 hrs. Compared with control group (Fig. 2C), the frequency of the cells undergoing apoptosis was less in the cell patch group (Fig. 2D) and the number of the apoptotic cells was smaller (*P* < 0.01; Fig. 2E). Consequently, the cell viability was higher (*P* < 0.01; Fig. 2F). The levels of *HIF‐1*α, *T*β*4*,* VEGF* and *SDF‐1* expression in MSCs in cell patch group were higher than that in control group, especially the level of *SDF‐1* expression. *CXCL14* expression in cell patch group was lower than that in control group (Fig. 2G).

### Improvement in the cardiac function after transplantation

**Figure 3:**
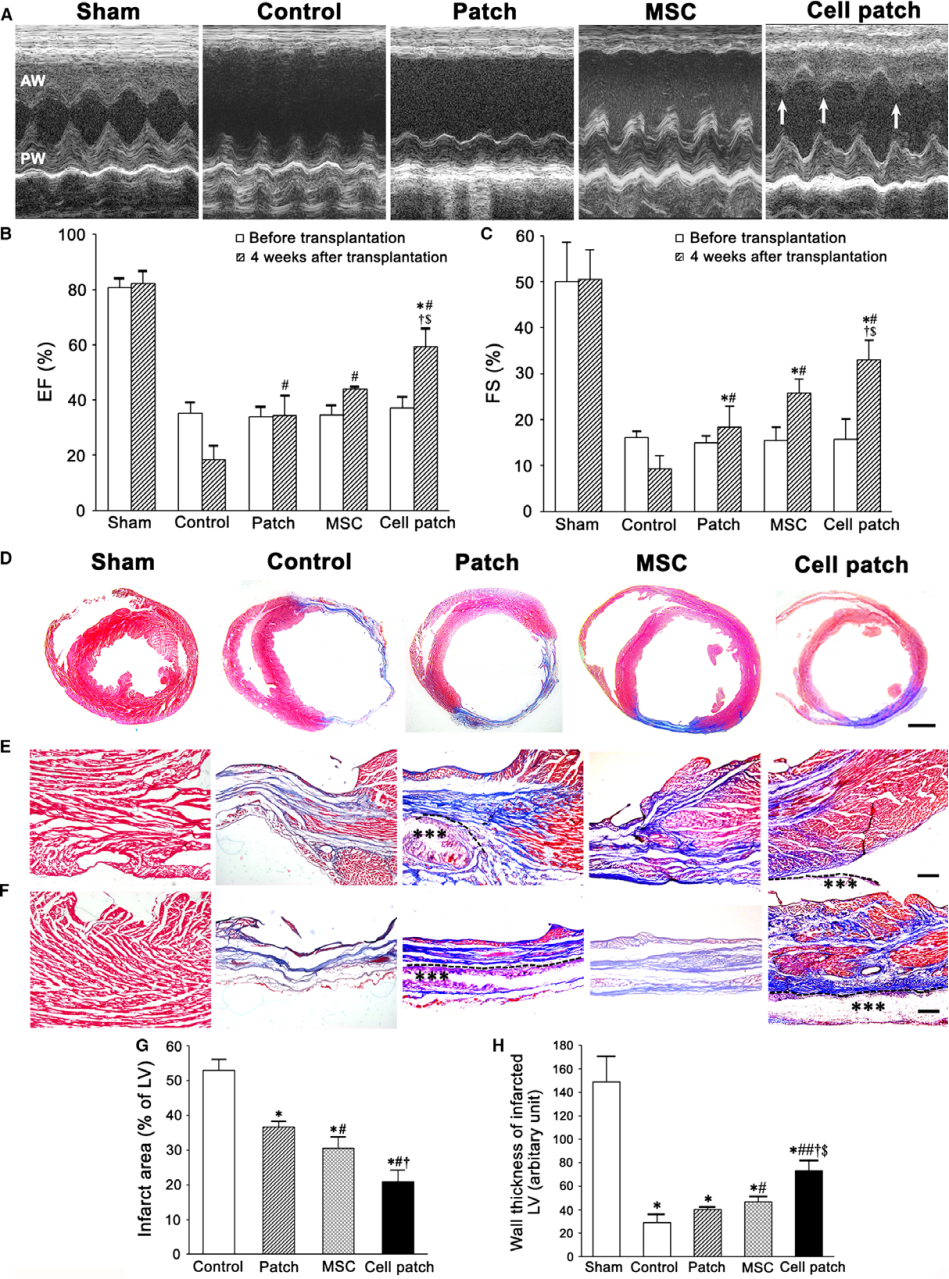
Restoration of LV function and structural changes in LV walls after transplantation. (**A**) Representative echocardiograms of LV free walls. LV contraction in the cell patch group was significantly improved (arrows). Compared with baseline, control, patch‐only and MSC‐only groups, EF (**B**) and FS (**C**) of cell patch group increase significantly (*n* = 6). *P < 0.01 *versus* baseline, ^#^ *P* < 0.01 *versus* control group; ^†^ *P* < 0.01 *versus* patch group; ^&^ *P* < 0.01 *versus* MSC group. (**D**) The transverse sections of the ventricles at the widest part of the infarct region. In control group, fibrosis is extensive, and LV wall is thin obviously in the infarct region (**F**), compared with control group, LV cavity is smaller, and LV wall in the infarct region is thicker in patch, MSC and cell patch groups. There are more myocardium (red) and less fibrous tissues (blue) in the peri‐infarct region (**E**) and infarct region (**F**) in MSC‐only and cell patch groups. Masson's trichrome staining. The dash line indicates the surface of the epicardium. Asterisks indicate the location of the patch. Scale bars: 2 mm (D), 100 μm (**E** and **F**). The graphs represent statistical results of the scar size (**G**) and thickness (**H**) of LV wall. *P < 0.01 *versus* control group; ^#^ *P* < 0.01 *versus* patch group; ^†^ *P* < 0.01 *versus* MSC group (**G**). *P < 0.01 *versus* sham group; ^#^ *P* < 0.05 *versus* control group; ^##^ *P* < 0.01 *versus* control group; ^†^ *P* < 0.01 *versus* patch group; ^$^ *P* < 0.01 *versus* MSC group (**H**). *n* = 9.

A series of echocardiographic examinations were conducted to evaluate LV function. EF and FS of healthy rats were 80.68 ± 3.44% and 49.93 ± 8.62%, respectively. At 1 week after LAD occlusion, the cardiac function of all rats was severely compromised, and the decline in cardiac function continued over the following 4 weeks in control group. In patch, MSC and cell patch groups, the cardiac function significantly improved (Fig. 3). Contraction of LV free wall was significantly enhanced in the cell patch group (Fig. 3A). FS in patch and MSC groups was higher than that in baseline (before transplantation; *P* < 0.01). EF in patch (34.26 ± 7.38) and MSC (44.02 ± 0.90) groups and FS in the two groups (18.39 ± 4.54 and 25.78 ± 3.07, respectively) increased significantly compared with control group (EF = 18.51 ± 5.03; FS = 9.29 ± 2.88; *P* < 0.01). Compared with baseline, control, patch, and MSC groups, the EF (59.34 ± 6.76) and FS (32.94 ± 4.33) in cell patch group increased significantly (Fig. 3B and C; *P* < 0.01).

### Morphological changes in the infarcted myocardium after transplantation

Presence of the scar in the anteroseptal and free walls of LV was observed in all groups after LAD occlusion. At 4 weeks after transplantation, the scar in control group extended to nearly whole free walls of LV. LV became dilated, and free LV wall was thin obviously. PCL/gelatin patch could restrict dilatation of the LV. After cell patch transplantation, dilatation and thinning of the LV wall was reduced greatly (Fig. 3D). Masson's staining of whole cardiac transverse sections showed that the myocardial tissue of the scarred area was replaced by fibrous tissue in control and patch groups, especially at the infarct region (Figs 3D and 3F). A little myocardial tissue at the infarct region was observed in MSC group. There was more myocardial tissue at the infarct region in cell patch group (Fig. 3F). The scar size of the infarct region in cell patch group (13.96 ± 1.79) was smaller than that in control (52.99 ± 3.17), patch (36.59 ± 1.61) and MSC (30.49 ± 3.32) groups (*P* < 0.01; Fig. 3G). Compared with control group, the scar size in patch and MSC groups became smaller (*P* < 0.01). The thickness of LV wall at the infarct region in MSC group increased significantly compared with that in control and patch groups (*P* < 0.01 and *P* < 0.05, respectively). The thickness of LV wall was greater in cell patch group (73.15 ± 8.87) than that in control (29.18 ± 7.25), patch (40.47 ± 2.11) and MSC (46.56 ± 5.05) groups (*P* < 0.01; Fig. 3H).

### Angiogenesis, lymphangiogenesis and myocardium regeneration after transplantation

**Figure 4:**
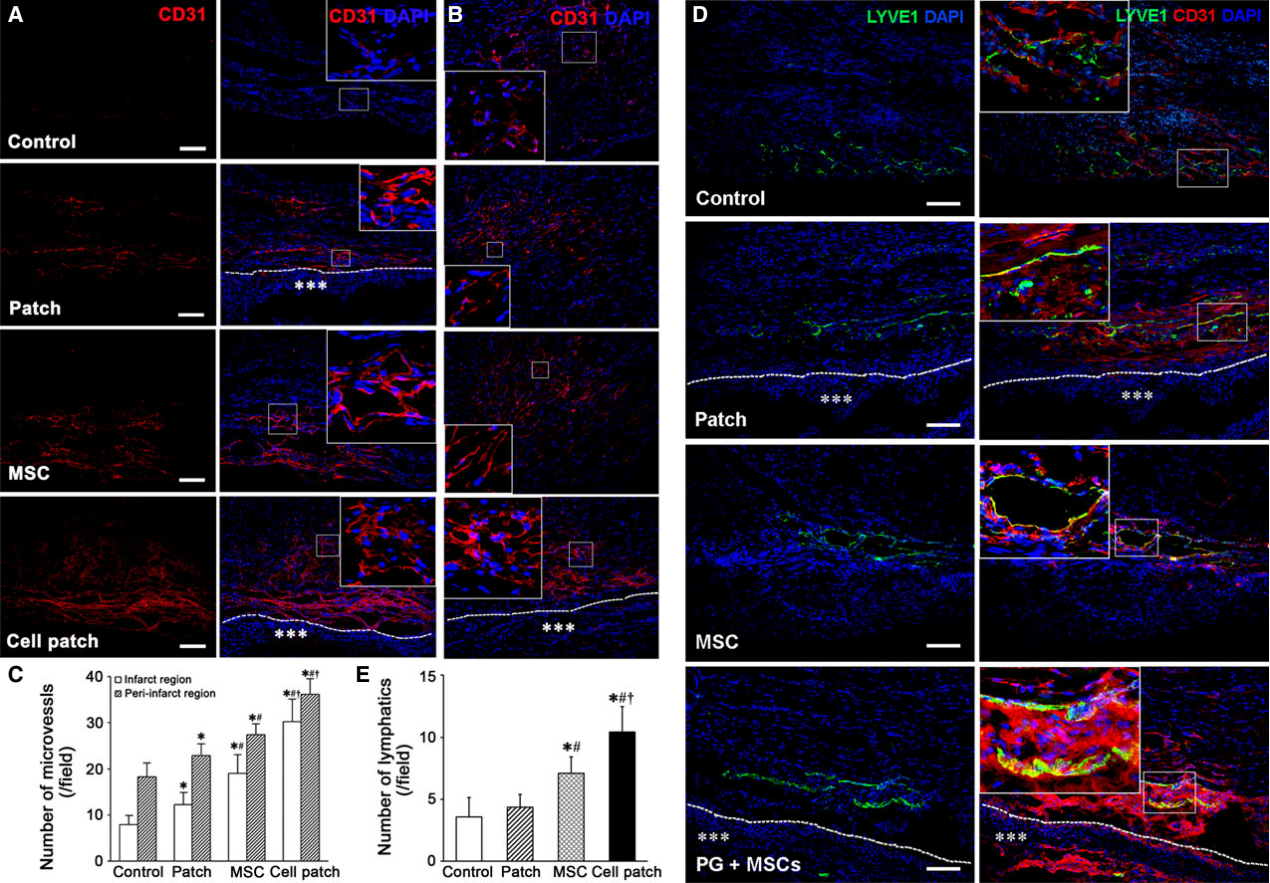
Microvessels and Lymphatic capillaries at the infarct and peri‐infarct regions. (**A** and **B**) CD31^+^ microvessels in the infarct and peri‐infarct regions. There are more microvessels in patch, MSC or cell patch groups than that in control group. The microvessels in cell patch group are more than that in patch‐ and MSC‐only groups. The large boxes are magnification of the small boxes. A line of dashes shows the surface of the epicardium. Asterisks indicate the patch. Scale bars represent 100 μm. (**C**) statistical results of microvessel density in the infarct and peri‐infarct regions. *P < 0.01 *versus* control group; ^#^ *P* < 0.01 *versus* patch group; ^†^ *P* < 0.01 *versus* MSC group. (**D**) LYVE‐1^+^ lymphatic capillaries increase in cell patch group compared with that in control, patch‐only and MSC‐only groups. The large boxes are magnification of the small boxes. A line of dashes shows the surface of the epicardium. Asterisks indicate the patch. Scale bars represent 100 μm. (**E**) statistical results of lymphatic density in the peri‐infarct regions. *P < 0.01 *versus* control group; ^#^ *P* < 0.01 *versus* patch group; ^†^ *P* < 0.01 *versus* MSC group. *n* = 9.

**Figure 5:**
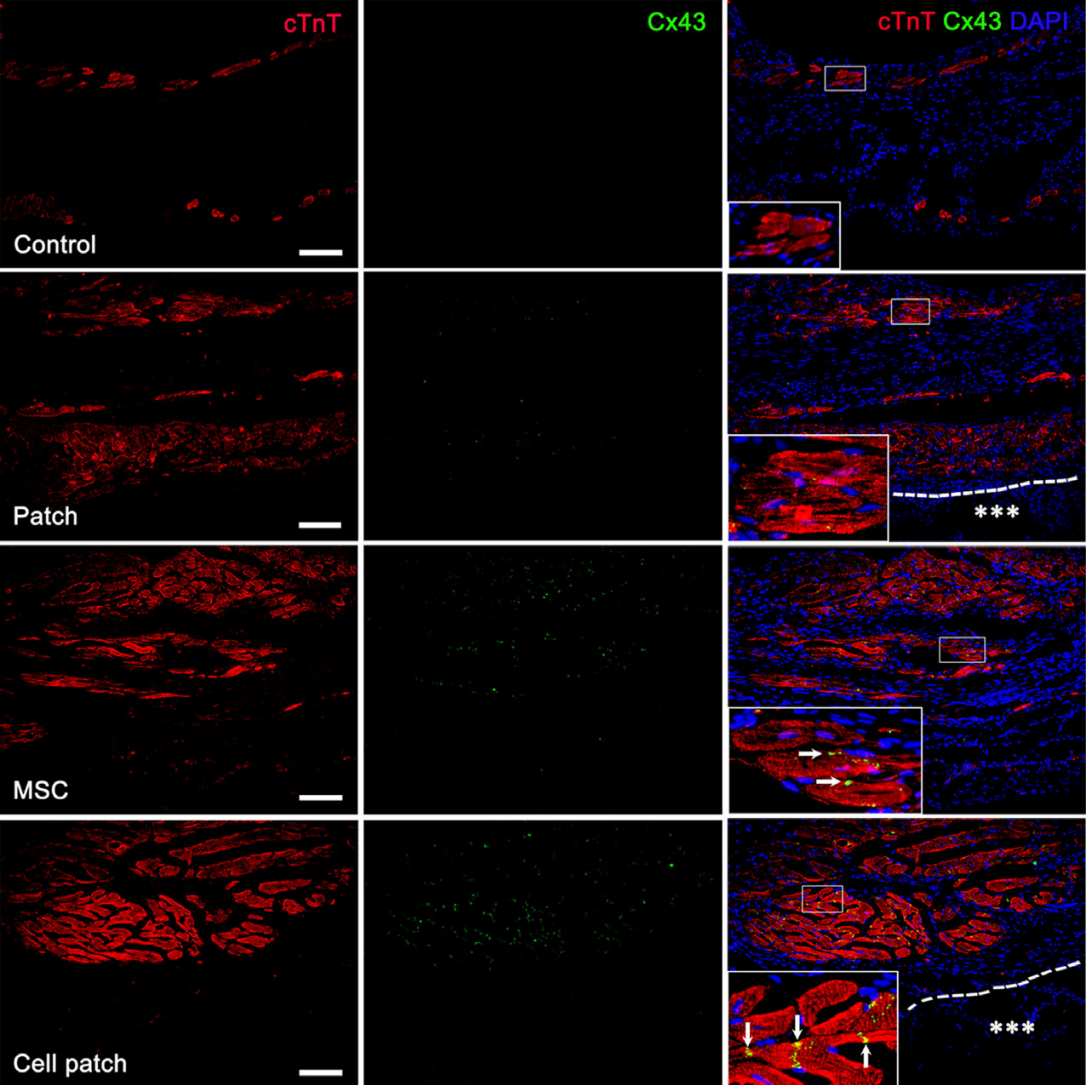
Myocardial regeneration at the infarct region. cTnT^+^ cardiomyocytes in the infarct region increase in MSC‐only and cell patch groups. Cx43 is expressed between the cardiomyocytes (arrows). The large boxes are magnification of the small boxes. A line of dashes shows the surface of the epicardium. Asterisks indicate the patch. Scale bars represent 100 μm.

Angiogenesis was determined by the number of CD31^+^ microvessels (20 × field) at the infarct region and peri‐infarct region at 4 weeks after transplantation. The microvessels at the infarct and peri‐infarct regions increased obviously in patch, MSC and cell patch groups, especially in cell patch group (Fig. 4A and B). Density of the microvessels at the infarct region in cell patch group (24.17 ± 4.93) was significantly greater than that in control (7.92 ± 2.02), patch (12.25 ± 2.60) and MSC (19.0 ± 4.09) groups (*P* < 0.01; Fig. 4C). Density of microvessels at the peri‐infarct region in cell patch group (35.08 ± 3.42) was greater than that in normal myocardial region (2.83 ± 1.11), and control (18.25 ± 3.02), patch (22.83 ± 2.62) and MSC (31.42 ± 2.31) groups (*P* < 0.01; Fig. 4C). LYVE‐1^+^ lymphatic capillaries were located mainly at the peri‐infarct region in all infarcted hearts. Compared with the non‐infarcted heart (normal or sham‐operated heart), the lymphatic capillaries in the infarcted heart increased significantly. The lymphatic capillaries at the peri‐infarct region in cell patch group were more than that in control, patch and MSC groups (Fig. 4D and E). To evaluate myocardial regeneration at the infarct region after transplantation, expression of cTnT and Cx43 was examined by immunostaining. At 4 weeks after cell patch transplantation, the fibrous tissue reduced and cTnT^+^ myocardial tissue increased significantly at the infarct region (Fig. 5). Expression of Cx43 could be observed between adjacent cardiomyocytes in MSC and cell patch groups (Fig. 5).

### Survival and differentiation of the engrafted cells

**Figure 6:**
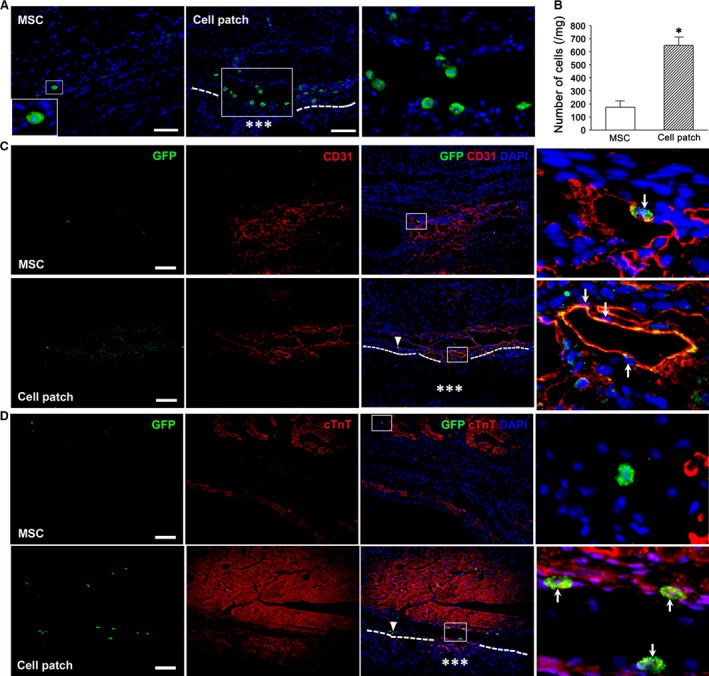
The survived and differentiated cells in the transplanted cells at 4 weeks. (**A**) the survived GFP^+^ MSCs in the infarct region. There are GFP^+^ cells in the epicardium and myocardium. GFP^+^ cells increase in cell patch group (the second and third panels). The third panel is magnification of the box in the second panel. (**B**) the survived *Sry* gene‐expressed MSCs at the infarct region. Compared with MSC‐only group, there are more survived cells in cell patch group *P < 0.01 *versus* MSC group. *n* = 3. (**C**) differentiation of the transplanted MSCs into endothelial cells. In cell patch group, some GFP^+^ cells express CD31, and these cells are located at the wall of the microvessels. (**D**) differentiation of the transplanted MSCs into cardiomyocytes. GFP^+^cTnT^+^ cells in cell patch group are mainly located in myocardium. Triangles show GFP^+^ cells, and arrows indicate GFP^+^CD31^+^ cells (**C**) and GFP^+^cTnT^+^ cells (**D**). A line of dashes shows the surface of the epicardium. Asterisks indicate the patch. The panels of the fourth row are magnification of the boxes in the panels of the third row (**C** and **D**). Scale bars represent 100 μm.

The survival of the transplanted MSCs was determined by GFP and *Sry* gene expression. At 4 weeks after transplantation, GFP^+^ cells in the cell patch group were more than those in the MSC group in the infarct region. GFP^+^ cells could be observed beneath the epicardium and in the myocardium (Fig. 6A). By quantifying the presence of the *Sry* gene, the number of the survived cells in cell patch group (649.76 ± 64.64 cell/mg) was greater than that in the MSC‐only group (174.0 ± 49.21 cell/mg; *P* < 0.05, Fig. 6B). To address whether differentiation of the transplanted cells towards endothelial cells or cardiomyocytes occurred, GFP and CD31 or cTnT double labelling was performed. Some GFP^+^ cells expressing CD31 were located at the wall of the microvessels, especially in cell patch group (Fig. 6C). There are some GFP^+^ cells expressing cTnT in cell patch group (Fig. 6D). GFP^+^cTnT^+^ cells were not observed in MSC‐only group (Fig. 6D).

### Activation of the epicardial cells and recruitment of endogenous c‐kit^+^ cells

**Figure 7:**
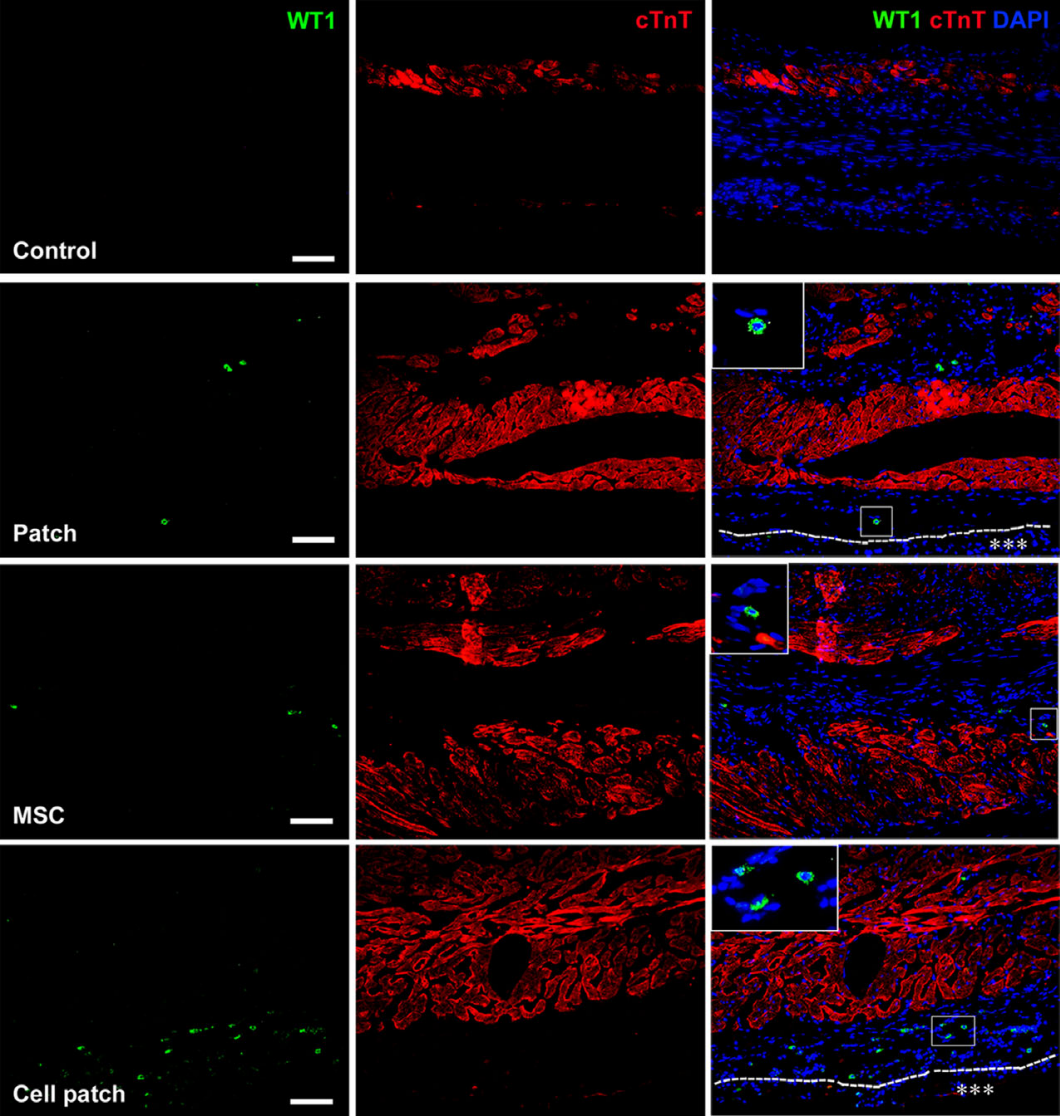
Distribution and differentiation of Wt1^+^ EPDCs in the infarct region of rats. Wt1^+^ EPDCs are located beneath the epicardium and in myocardium of rats at 4 weeks after transplantation. The cells are more in the infarct region in cell patch group than that in patch‐ and MSC‐only groups. The large boxes are magnification of the small boxes. A line of dashes shows the surface of the epicardium. Asterisks indicate the patch. Scale bars represent 100 μm.

**Figure 8:**
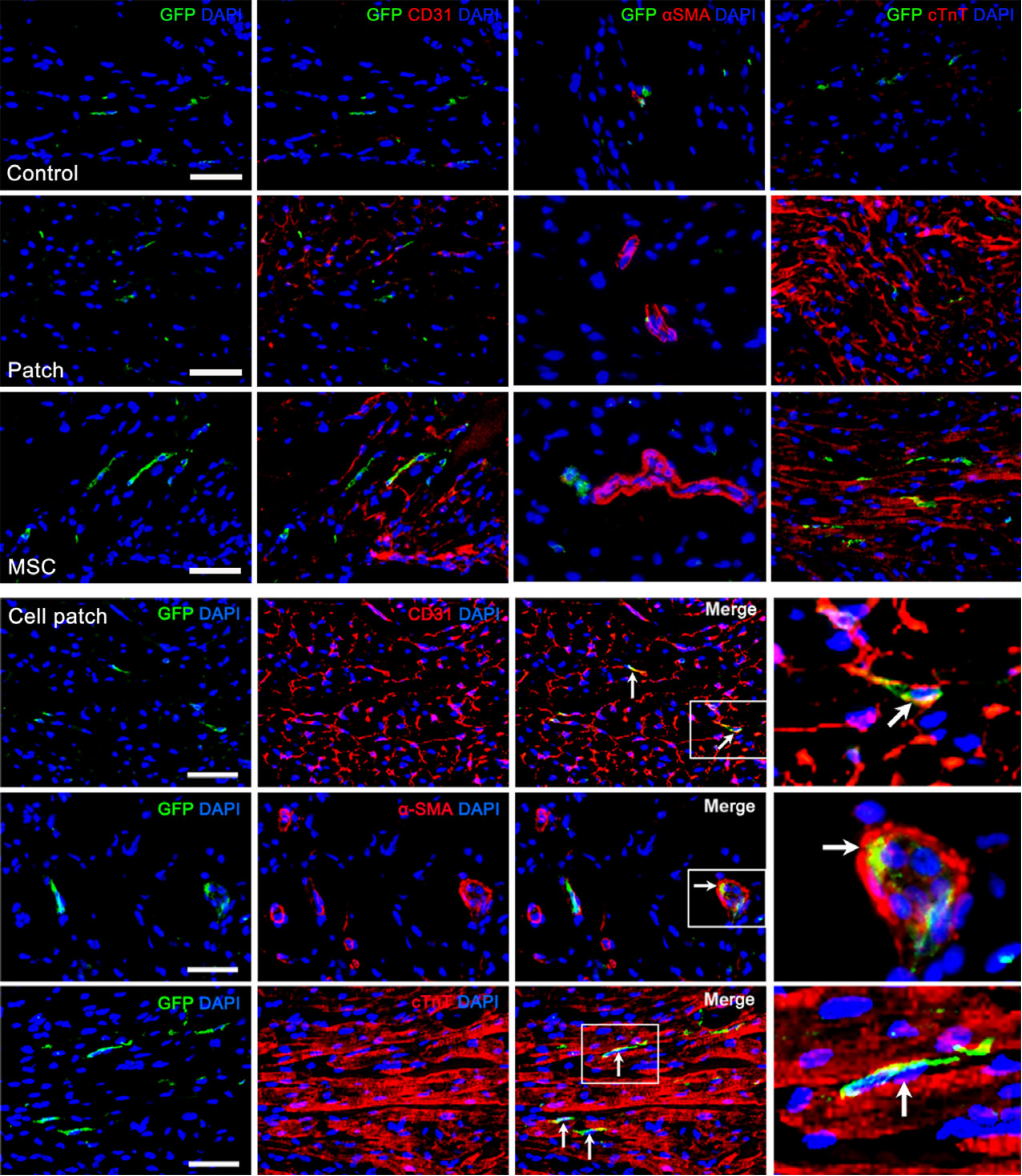
Distribution and differentiation of Wt1^+^ EPDCs in the infarct region of transgenic mice. GFP^+^ (Wt1^+^) cells migrate into myocardium of the transgenic mice hearts at 4 weeks after transplantation. In the control and patch groups, a few GFP^+^ cells were observed. There are more GFP^+^ cells in myocardium in MSC and cell patch groups. In the cell patch group, some GFP^+^ cells express CD31, α‐SMA or cTnT (arrows). GFP^+^CD31^+^ cells and GFP^+^α‐SMA^+^ cells are located at the wall of microvessels, while GFP^+^cTnT^+^ cells adhere on adjacent cardiomyocytes. The fourth panel is magnification of the boxes in the third panel. Scale bars represent 100 μm.

**Figure 9:**
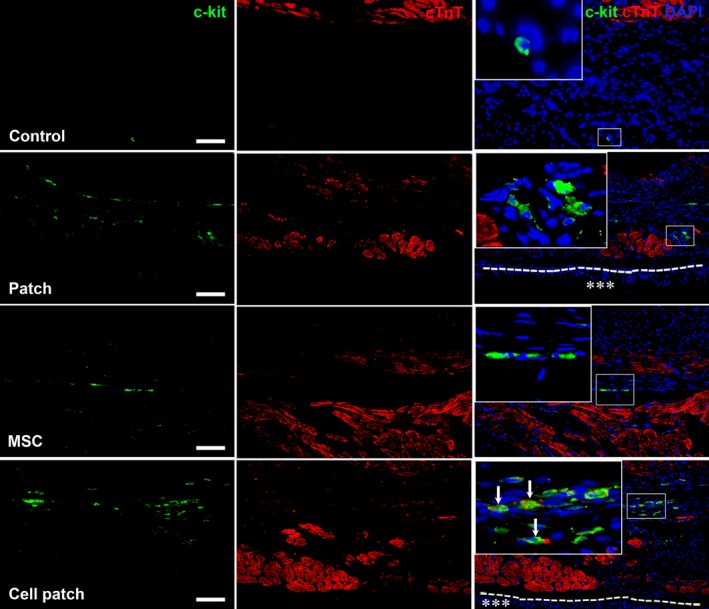
Distribution of c‐kit^+^ cells in the infarct region. At 4 weeks after transplantation, c‐kit^+^ cells in the infarct region increase significantly in cell patch group compared with patch‐ and MSC‐only groups. Some c‐kit^+^ cells express cTnT weakly. The dash line indicates the surface of the epicardium. Asterisks indicate the location of the patch. The large boxes show magnified views of the small boxes. Scale bars represent 100 μm.

Earlier studies have reported that epicardial‐activated Wt1^+^ cells give rise to EPDCs in the heart 20, 21. In rat MI models, EPDCs were observed to localize beneath the epicardium and within the myocardium of the infarct region at 4 weeks after transplantation. We show that there are more EPDCs in cell patch group than that in patch or MSC‐only groups (Fig. 7). In control group, Wt1^+^ EPDCs were not observed (Fig. 7). The distribution of EPDCs in mice is similar to those in rat, but there are more GFP^+^ cells in mouse myocardium. The co‐expression of GFP and CD31, α‐SMA or cTnT, indicating cardiovascular differentiation of EDPCs, was observed more frequently after cell patch transplantation. GFP^+^CD31^+^ and GFP^+^α‐SMA^+^ cells were found within the luminal wall of microvessels. GFP^+^cTnT^+^ cells were located within a group of cardiomyocytes (Fig. 8). In rat MI models, there were more c‐kit^+^ cells in the infarct region of patch, MSC and cell patch groups than in control group (Fig. 9). Compared with patch or MSC‐only groups, the cell patch group shows significant increase in c‐kit^+^ cells. Some c‐kit^+^ cells in cell patch group expressed a low level of cTnT (Fig. 9). To distinguish endogenous c‐kit^+^ cells in the heart from c‐kit^+^ cells within the transplanted MSCs, double staining of c‐kit and GFP was performed. We found no double c‐kit^+^GFP^+^ cells in cell patch group, supporting the endogenous origin of the c‐kit^+^ cells and not from the transplanted MSCs (data not shown).

### Expression of *HIF‐1*α, *T*β*4*,* VEGF*,* SDF‐1* and *CXCL14* in the infarcted myocardium

**Figure 10:**
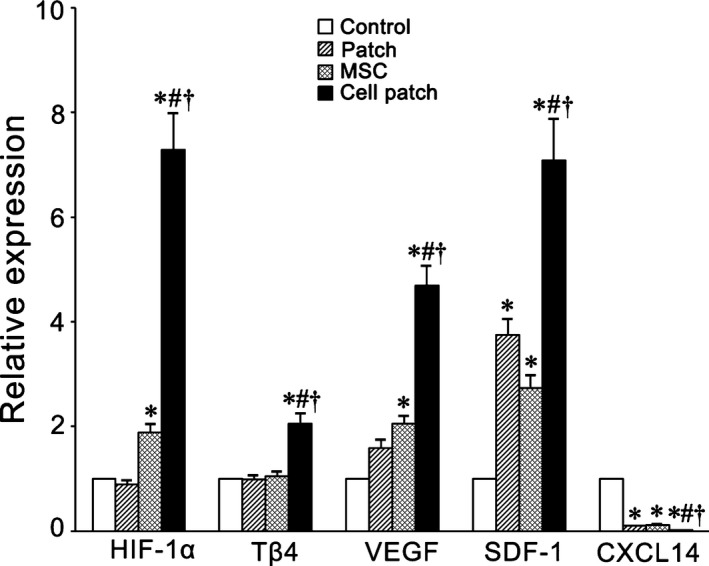
Expression of *HIF‐1*α, *T*β*4*,* VEGF*,* SDF‐1* and *CXCL14* in infarcted myocardial tissue. At 7 days after transplantation, expression of *HIF‐1*α, *T*β*4*,* VEGF* and *SDF‐1* in the infarcted myocardial tissue was higher significantly in cell patch group than in control, patch‐only and MSC‐only groups. However, expression level of *CXCL14* in cell patch group was lower significantly than in control, patch‐only and MSC‐only groups. All expression levels were normalized to that in the control group, which was assigned a value of 1.0. *P < 0.05 *versus* control group; ^#^ *P* < 0.05 *versus* patch‐only group; ^†^ *P* < 0.05 *versus* MSC‐only group. *n* = 6.

In the infarcted myocardium, expression of *HIF‐1*α, *T*β*4*,* VEGF* and *SDF‐1* increased at 7 days after transplantation. Compared with control, patch‐only or MSC‐only groups, the cell patch group showed significantly higher expression of these genes, especially expression of *HIF‐1*α and *SDF‐1*. *SDF‐1* expression in patch‐only group was higher than that in control group. Expression of *HIF‐1*α, *VEGF* and *SDF‐1* in MSC‐only group was higher than that in control group. On the contrary, expression level of *CXCL14* in patch, MSC and cell patch groups was significantly lower than that in control. Expression of *CXCL14* in cell patch group was lower than in patch‐ and MSC‐only groups (Fig. 10).

## Discussion

The present study demonstrates that epicardial transplantation of MSC‐loaded PCL/gelatin patch is an effective therapeutic strategy for repairing the infarcted myocardium and improving cardiac function. Both the transplanted cells and EPDCs activated by the cell patch contribute to repair of the infracted myocardium.

Our data show that PCL/gelatin patch provides a suitable microenvironment for engraftment and growth of MSCs. Because of biocompatible degradation properties, PCL is approved for use in human beings by the US Food and Drug Administration 22. Gelatin is a mixture of peptides and proteins produced by partial hydrolysis of collagen extracted from animals. We found that MSCs adhere and spread well on nanofibres of PCL/gelatin patch. Application of the patch did not cause arrhythmias and inflammatory responses. It is worth noting that the patch may be degraded by 6 months (data not include). The pores of the nanofibrous patch permit the surrounding microvessels to grow into patch easily 17, which maintains better survival and growth of the transplanted MSCs. Optimization of the size of the pores in the patch will help to maximize vascularization within the implant and minimizes fibrosis around it 3. Moreover, the porous patch will allow pericardial fluid to stimulate the survival of the loaded MSCs, as the pericardial fluid from patients with ischaemic heart disease is enriched in growth factors such as VEGF and bFGF released from pericardial and epicardial cells as well as from the myocardium 23. In hypoxic and serum‐deprived conditions, the patch was shown to maintain the survival and viability of the seeded MSCs. In addition, our data *in vivo* show that the epicardial patch can enhance MSC engraftment when compared to direct intramyocardium injection of MSCs. Pre‐adaption of MSCs with hypoxia and ischaemia on the epicardium appears to improve survival and differentiation of the cells after their migration into the hostile environment of the infarcted myocardium. Hypoxic precondition‐induced autophagy may be beneficial for cell survival in a local hypoxic environment 24. Therefore, we suggest that epicardial transplantation of MSC‐loaded PCL/gelatin patch is an optimal strategy for enhancing MSC engraftment.

To address the molecular mechanism of the benefit by MSC‐loaded patch, we found that cell patch transplantation can contribute to reduction in the cardiac remodelling by restricting dilation of the infarcted LV wall and maintaining LV mechanical property. We also found that endothelial cells differentiated from the engrafted MSCs incorporate into the wall of the new‐formed microvessels. The regenerated cardiomyocytes from MSCs were found to connect with each other and electrically couple with host cardiomyocytes by Cx43^+^ expression at the junction (intercalated disc), which is a crucial structure for establishing sustained synchronous contraction of the myocardium.

Interestingly, we found that epicardial transplantation of MSC‐loaded patch can effectively activate the epicardium and promote differentiation of the activated EPDCs towards cardiovascular cells. The epicardium has been reported to play an active role during normal cardiomyogenesis 25, while it becomes quiescent after birth 26. Although the mammalian epicardium is activated after MI, the number of the activated cells is insufficient for myocardium repair 27, 28. MSCs secrete anti‐inflammatory, immunosuppressive, anti‐apoptotic and proangiogenic factors 29, 30. MSC‐conditioned medium increases capillary density and preserves cardiac function 31. Recent clinical trials have suggested that secretion of paracrine factors may be the underlying mechanism responsible for the improvement in outcomes 4. In this study, we found that epicardial transplantation of MSC‐loaded patch enhances sustained production of Tβ4, HIF‐1α and VEGF by the engrafted cells and infarcted cardiac tissue and is more effective for simulating the epicardium compared with intramyocardial MSC transplantation. After epicardial transplantation of the cell patch for 4 weeks, the activated Wt1^+^ EPDCs increase significantly and migrate into subepicardium and myocardium. These cells were found to differentiate into endothelial cells, vascular smooth muscle cells and cardiomyocytes. In Wt1^CreERT2/+^,R26^mTmG^ mice, lineage‐traced EPDCs express CD31, α‐SMA and cTnT within myocardium of the infarcted region along with GFP.

In this study, we also showed that the cell patch has a positive effect on recruit endogenous c‐kit^+^ cells. Compared with intramyocardial transplantation group, epicardial transplantation of the cell patch enhanced recruitment of c‐kit^+^ cells into the infarcted myocardium. At 4 weeks after transplantation, endogenous c‐kit^+^ cells were observed at the infarcted region, while among the transplanted MSCs, c‐kit^+^ cells were not detected. SDF‐1 and VEGF released by the engrafted MSCs and infarcted cardiac tissue are responsible for mobilization of c‐kit^+^ cells from heart itself and from bone marrow. Transportation of extracellular microvesicles may be accounted for paracrine of the factors 32. *CXCL14* expression was found to be down‐regulated after cell patch transplantation. CXCL14 specifically binds to CXCR4 with high affinity and inhibits the CXCL12‐mediated cell migration 33. Moreover, CXCL14 is a potent inhibitor of angiogenesis 34. Decrease in *CXCL14* expression in this study promotes migration of c‐kit^+^ cells as well as angiogenesis. c‐kit^+^ stem cells increase 20‐fold after intramyocardial MSC injection 33. Taking together, this study suggests that MSC engraftment on the epicardium favours induction of c‐kit^+^ stem cells in nearby tissue. Moreover, the factors released from the engrafted MSCs may be responsible for recruiting these stem cells. In addition, some c‐kit^+^ cells express cTnT after cell patch transplantation, suggesting that endogenous c‐kit^+^ cells may undergo early steps of differentiation into cardiomyocytes.

Cardiac lymphatic vessels drain excess fluid from the extracellular spaces and transport leucocytes and antigen‐presenting cells from inflammatory tissue. After MI, the number and diameter of cardiac lymphatic vessels increase. Lymphangiogenesis occurs after MI 35. Enhancement of lymphangiogenesis may reduce myocardial oedema and cardiac fibrosis, promoting improvement in cardiac function 36, 37. Our data show that lymphatic capillaries in the infarcted heart increased in cell patch group than that in patch‐ or MSC‐only groups. Although beyond the scope of the current paper, we believe the effect of cardiac cell patch on lymphangiogenesis should undergo further investigation.

In summary, PCL/gelatin patch restricts effectively ventricular dilation and attenuate adverse remodelling of ventricular wall with mechanical property. As nanofibrous scaffold and component of extracellular matrix, PCL/gelatin patch favours adhesion and growth of the seeded MSCs. Epicardial transplantation of MSCs loading by PCL/gelatin patch may protect the cells to survive although the local epicardium is in condition of hypoxia. MSC‐released cytokines enhance activation of the epicardium and recruitment of endogenous c‐kit^+^ cells *via* paracrine mechanism. Epicardial transplantation of MSC‐loaded PCL/gelatin patch can effectively promote myocardium regeneration and angiogenesis. Therefore, this novel cardiac patch is a desirable therapy for repairing the infracted myocardium.

## Disclosures

These authors declare no conflict of interest.
